# Editorial: Advances on molecular mechanisms regulating reproduction - from gametogenesis to fertilization

**DOI:** 10.3389/fmolb.2026.1850838

**Published:** 2026-04-23

**Authors:** David F. Carrageta, Anna Ptak, Raquel L. Bernardino

**Affiliations:** 1 UMIB – Unit for Multidisciplinary Research in Biomedicine, ICBAS – School of Medicine and Biomedical Sciences, University of Porto, Porto, Portugal; 2 ITR – Laboratory for Integrative and Translational Research in Population Health, Porto, Portugal; 3 Laboratory of Physiology and Toxicology of Reproduction, Institute of Zoology and Biomedical Research, Faculty of Biology, Jagiellonian University, Krakow, Poland

**Keywords:** hormonal regulation, inflammation, metabolism, reproduction, signaling pathways

Infertility, defined as the inability to conceive after 12 months of regular unprotected intercourse, is a major global health challenge affecting one in six couples of reproductive age (WHO, 2023). It is a multifactorial condition, attributed to male, female, or combined factors (Agarwal et al., 2021; Liu et al., 2025). However, a significant percentage of cases are classified as idiopathic (Carson and Kallen, 2021), mainly due to our limited understanding of the processes underlying human reproduction.

Gametogenesis and gamete function are very complex processes, with time- and location-dependent regulation by an intricate network of hormonal, metabolic, genetic, and epigenetic factors (Guerra-Carvalho et al., 2022; Longo et al., 2025). Since animal models do not accurately reflect human reproductive biology (Cunha et al., 2019; Carrageta et al., 2022), and experimental procedures involving human fertility are invasive and ethically challenging (Iltis et al., 2023; Anifandis et al., 2022), progress in this field remains particularly difficult. A better understanding of the molecular mechanisms underlying human fertility is essential not only for comprehending physiological processes but also for the development of targeted interventions that can either treat human infertility or lead to the development of novel contraceptives.

The goal of this Research Topic was to compile original and review studies offering new perspectives on the molecular mechanisms governing reproduction. Among the many factors that regulate reproduction, inflammatory processes were the focus of the great majority of the research included in this Research Topic.

Harrison et al. investigated how human seminal fluid modulates immunity to protect sperm in the female reproductive tract. The authors demonstrated that the human seminal fluid contains abundant soluble CD52 bound to the danger-associated molecular pattern protein high mobility group box 1 (HMGB1), which indirectly suppresses T-cell responses via the immune-suppressive receptor Siglec-7. It was also reported that seminal fluid strongly inhibits the proliferation of natural killer (NK) cells, an effect that is lost when CD52 is immunodepleted. Notably, the immunosuppressive effect was primarily associated with the soluble fraction rather than with prostasomes. Overall, this study identified soluble CD52 as a key mediator of the immunosuppressive activity of human seminal fluid, potentially protecting sperm from immune responses in the female reproductive tract.

Correnti et al. used an untargeted HPLC-MS metabolomics approach to explore the metabolic signature of human seminal fluid in cases of teratozoospermia (i.e., a high prevalence of abnormal sperm morphology). Compared to normozoospermia, the seminal fluid of donors with teratozoospermia exhibited a significantly altered metabolome, characterized by 14 metabolites significantly altered and related to inflammation and oxidative stress. Among these, O-acetyl-L-serine, creatine, and histidine were identified as candidate biomarkers for teratozoospermia and exhibited a robust discriminatory power following ROC analysis. Overall, these findings suggest that inflammation and oxidative stress may be related to the pathophysiology of teratozoospermia, which provides a foundation for anti-inflammatory and antioxidant therapies in the management of these patients. Using an LC-MS/MS proteomics approach, Mierzejewski et al. investigated how lipopolysaccharide (LPS)-induced inflammation affects the porcine corpus luteum during the mid-luteal phase. After 24 h of *in vitro* LPS exposure, 12 differently regulated proteins were identified, including increased UTP-glucose-1-phosphate uridylyltransferase (UGP2) and succinate-CoA ligase GDP-forming subunit beta (SUCLG2), suggesting altered glucose metabolism and Krebs cycle activity, and increased NAD(P)-dependent steroid dehydrogenase-like (NSDHL), suggesting an impact on corpus luteum steroidogenesis. Consistent with these findings, glucose content was decreased in the LPS-exposed corpus luteum, while progesterone increased. Together, these results show that the corpus luteum responds to inflammatory stimuli, leading to an altered metabolic and steroidogenic function with potential consequences for embryo implantation and early pregnancy support.

Turgay et al. analyzed the inflammatory markers neutrophil gelatinase-associated lipocalin (NGAL) and matrix metalloproteinase-9 (MMP-9) as candidate biomarkers for endometrioma. By comparing the serum levels of patients with unexplained infertility (n = 45) to those of patients with diagnosed endometrioma (n = 45), the authors found increased NGAL levels and decreased MMP-9 levels in the endometrioma group, resulting in a markedly higher MMP-9/NGAL ratio. Postoperative analysis showed decreased MMP-9 levels in endometrioma patients, aligning the MMP-9/NGAL ratio with that of the unexplained infertility group. ROC analysis revealed an excellent discriminatory performance for the MMP-9/NGAL ratio (AUC = 0.898), with a proposed cutoff of >1.75 yielding 86.1% sensitivity and 84% specificity for diagnosing endometrioma. Overall, the MMP-9/NGAL ratio appears to be a useful complementary tool to ultrasonography in diagnosing endometrioma.

Infertility-related genetic factors were also covered in this Research Topic. Kazama et al. studied the role of transducin-like enhancer of split 6 (TLE6), a key component of the subcortical maternal complex (SCMC), in male reproductive function using a *Tle6* heterozygous (*Tle6* ^*+/−*^) C57BL/6N mouse model generated using CRISPR-Cas9 technology. Although litter size was similar to that of wild-type mice, the offspring genotype proportion deviated from Mendelian laws. Embryos derived from *Tle6* ^*+/−*^ mice showed a normal developmental rate, which led the authors to hypothesize that these mice have poorer sperm quality. Despite the absence of morphological changes in the seminiferous tubules, *Tle6* ^*+/−*^ mice exhibited a reduced sperm count, a marked decrease in highly motile sperm, and an increased incidence of morphological defects in the sperm’s head. Immunofluorescence staining revealed that TLE6 localizes in the midpiece of mice’s sperm, suggesting a functional role in motility and potentially in energy production. These findings indicate that TLE6 may influence spermatogenesis and sperm function, highlighting the need for future human studies.

Finally, Zhao et al. conducted a critical narrative review on mitophagy, a selective process that preserves mitochondrial quality by removing damaged mitochondria, and its role in folliculogenesis, oocyte maturation, fertilization, and embryo implantation. The review highlights that dysregulated mitophagy affects stromal cell survival and migration, thereby contributing to endometriosis, while also being implicated in polycystic ovary syndrome, where excessive mitophagy in granulosa cells leads to impaired folliculogenesis, in primary ovarian insufficiency, and in ovarian aging. Interestingly, the authors emphasize that mitophagy can act bidirectionally, depending on the cellular redox state, and may therefore be either protective or detrimental to reproductive cells. Therapeutic strategies are discussed, including the potential use of melatonin and zinc, along with natural products used in traditional Chinese medicine.

The editors of this Research Topic thank all submitting authors for their work. They would also like to thank all reviewers for reading and commenting on submitted manuscripts. As the editorial team, we hope that this Research Topic will be useful for future studies in Reproductive Medicine.
